# Crystal structure of the co-crystal of 5-amino­isophthalic acid and 1,2-bis(pyridin-4-yl)ethene

**DOI:** 10.1107/S2056989016005259

**Published:** 2016-04-08

**Authors:** Scott C. McGuire, Steven C. Travis, Daniel W. Tuohey, Thomas J. Deering, Bob Martin, Jordan M. Cox, Jason B. Benedict

**Affiliations:** a 764 Natural Sciences Complex, Buffalo, 14260-3000, USA; b 345 Natural Sciences Complex, Buffalo, 14260-3000, USA; c 771 Natural Sciences Complex, Buffalo, 14260-3000, USA

**Keywords:** crystal structure, 5-aminoisophthalic acid, 5AIA, 1,2-bis(pyridin-4-yl)ethene (BE), co-crystal, hydrogen bonding

## Abstract

In the title 1:1 co-crystal, C_12_H_10_N_2_·C_8_H_7_NO_4_, the bi­pyridine moiety shows whole-mol­ecule disorder over two sets of sites in a 0.588 (3): 0.412 (3) ratio. In the crystal, the components form hydrogen-bonded sheets linked by N—H⋯O and O—H⋯N inter­actions, which stack along the *a* axis. A comparison to a related and previously published co-crystal of 5-amino-isophthalic acid and the shorter 4,4′-bipryidine is presented.

## Abstract 2

The supra­molecular structure of the title 1:1 co-crystal consists of (100) sheets linked by O—H⋯N and N—H⋯O hydrogen bonds.

## Chemical context   

5-Amino-isophthalic acid (5AIA) is an emerging secondary building unit for a wide variety of metal–organic frameworks (MOFs). (Zeng *et al.*, 2009 ▸; Wang *et al.*, 2011 ▸; Cox *et al.*, 2015 ▸) This compound is also a convenient precursor for the synthesis of azo-derivatized framework ligands, a key component in the rapidly evolving field of photochromic MOFs. (Brown *et al.*, 2013 ▸; Castellanos *et al.*, 2016 ▸; Walton *et al.*, 2013 ▸; Patel *et al.*, 2014 ▸). Similarly, 1,2-bis(pyridin-4-yl)ethene (BE) is also commonly used in MOF synthesis; however, it is routinely used in co-crystal engineering as well (Kongshaug & Fjellvag, 2003 ▸; MacGillivray *et al.*, 2008 ▸; Desiraju, 1995 ▸) The 5AIA–BE co-crystal presented herein was produced as part of an undergraduate physical chemistry laboratory experiment developed by Jason Benedict.
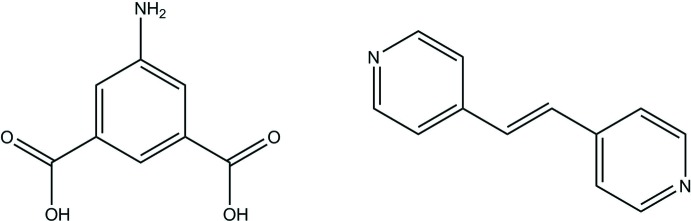


Recently, the co-crystal structure of 5AIA and 4,4′-bi­pyridine (BP), a shorter analogue of BE, was reported (Zhang *et al.*, 2009 ▸). Unlike many MOFs in which different length linkers lead to isorecticular structures (Eddaoudi *et al.*, 2002 ▸), the 5AIA–BP co-crystal exhibits several notable similarities and differences when compared to 5AIA–BE. As shown in Figs. 4, 5AIA forms hydrogen bonds with two 5AIA mol­ecules and two BP mol­ecules. The 5AIA–BP inter­actions and one of the 5AIA–5AIA inter­actions are similar to those found in 5AIA–BE. The remaining 5AIA–5AIA inter­action in 5AIA–BP consists solely of an N(amine)–H⋯OH hydrogen bond, as opposed to the N(amine)—H⋯O=C inter­action found in 5AIA–BP. Inter­estingly, this results in a total of five hydrogen bonds in the 5AIA–BP structure compared to the six hydrogen bonds observed in 5AIA–BE.

## Structural commentary   

**Figure 1:**
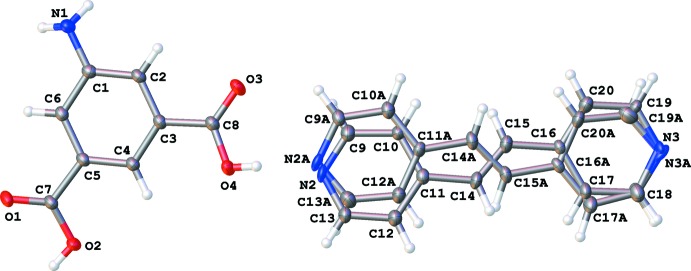
The asymmetric unit of the title compound, showing the numbering scheme. Displacement ellipsoids are shown at the 50% probability level.

The 5AIA–BE co-crystal crystallizes with one mol­ecule of 5AIA and one mol­ecule of BE in the asymmetric unit (Fig. 1 ▸). Both mol­ecules are effectively planar in the solid state (r.m.s. deviation for 5AIA = 0.155 Å). The BE moiety shows whole mol­ecule disorder over two sets of sites, consistent with a local *C*2 rotation about the long axis of the mol­ecule. The occupancy of the major and minor components was refined to be 0.588 (3) and 0.412 (3), respectively.

## Supra­molecular features   

**Figure 2:**
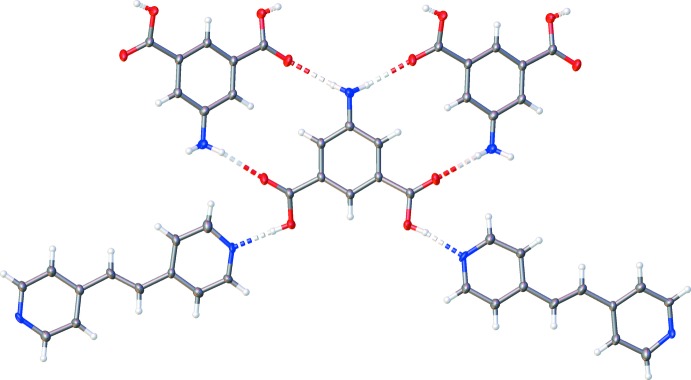
Diagram illustrating the hydrogen-bonding inter­actions present in the two-dimensional sheets found in the 5AIA–BE co-crystal.

**Figure 3:**
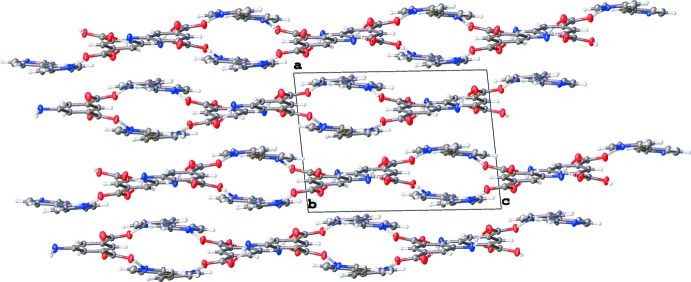
View down [001] showing the (100) sheets in the extended structure of the title compound.

In this structure, the 5AIA mol­ecule forms hydrogen bonds to both itself and the BE moiety, forming extended sheets (Table 1 ▸ and Fig. 2 ▸). The 5AIA–5AIA inter­actions consist of N(amine)—H⋯O=C hydrogen bonds where each 5AIA makes two hydrogen bonds with two neighboring 5AIA mol­ecules. The 5AIA–BE inter­action consists of an O—H⋯N(pyrid­yl) hydrogen bond such that each 5AIA makes one hydrogen bond with two neighboring BE mol­ecules. The sheets formed by these inter­actions stack along the the *a* axis to produce a layered structure (Fig. 3 ▸).

## Database survey   

**Figure 4:**
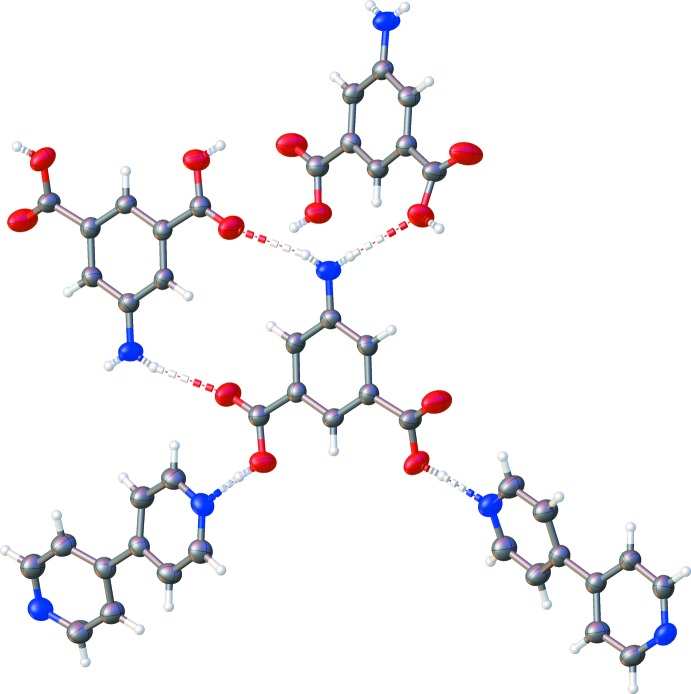
Diagram illustrating the hydrogen bonding inter­actions present in the previously reported 5AIA–BP co-crystal.

Recently, the co-crystal structure of 5AIA and 4,4′-bi­pyridine (BP), a shorter analogue of BE, was reported (Zhang *et al.*, 2009 ▸). Unlike many MOFs in which different length linkers lead to isorecticular structures (Eddaoudi *et al.*, 2002 ▸), the 5AIA–BP co-crystal exhibits several notable similarites and differences when compared to 5AIA–BE. As shown in Figs. 4 ▸, 5AIA forms hydrogen bonds with two 5AIA mol­ecules and two BP mol­ecules. The 5AIA–BP inter­actions and one of the 5AIA–5AIA inter­actions are similar to those found in 5AIA–BE. The remaining 5AIA–5AIA inter­action in 5AIA–BP consists solely of an N(amine)—H⋯OH hydrogen bond, as opposed to the N(amine)—H⋯O=C inter­action found in 5AIA–BP. Inter­estingly, this results in a total of five hydrogen bonds in the 5AIA–BP structure compared to the six hydrogen bonds observed in 5AIA–BE.

## Synthesis and crystallization   

Solid BE (0.0119 g, 6.53 × 10^−5^ mol) and 5AIA (0.0109 g, 6.02 × 10^−5^ mol) were added to a 25 ml scintillation vial. To this was added approximately 15 ml of ethyl acetate followed by gentle heating. An additional 2 ml of methanol was added and all remaining solids dissolved. The loosely capped vial was then placed into a dark cabinet. After two weeks, yellow block-shaped crystals of the title compound suitable for single-crystal X-ray diffraction measurements were obtained.

## Refinement   

Crystal data, data collection and structure refinement details are summarized in Table 2 ▸. Heteroatom hydrogen atoms were located in difference electron-density maps and freely refined. Hydrogen atoms attached to carbon atoms were refined using riding models with C—H = 0.95 Å and *U*_iso_(H) = 1.2*U*_eq_(C). The BE was found to be disordered over two sets of sites in a 0.588 (3): 0.412 (3) ratio.

## Supplementary Material

Crystal structure: contains datablock(s) I. DOI: 10.1107/S2056989016005259/hb7561sup1.cif

Structure factors: contains datablock(s) I. DOI: 10.1107/S2056989016005259/hb7561Isup2.hkl

CCDC reference: 1471029

Additional supporting information:  crystallographic information; 3D view; checkCIF report

## Figures and Tables

**Table 1:** Hydrogen-bond geometry (Å, °)

*D*—H⋯*A*	*D*—H	H⋯*A*	*D*⋯*A*	*D*—H⋯*A*
N1—H1*A*⋯O1^i^	0.899 (17)	2.062 (17)	2.9540 (13)	171.0 (15)
N1—H1*B*⋯O3^ii^	0.894 (17)	2.157 (17)	3.0500 (13)	178.6 (13)
O2—H2⋯N3^iii^	0.989 (19)	1.70 (2)	2.688 (8)	173.4 (18)
O2—H2⋯N3*A* ^iii^	0.989 (19)	1.63 (2)	2.619 (12)	177 (2)
O4—H4⋯N2^iv^	0.98 (2)	1.72 (2)	2.702 (7)	173.2 (19)
O4—H4⋯N2*A* ^iv^	0.98 (2)	1.59 (2)	2.566 (11)	175 (2)

Symmetry codes: (i) 
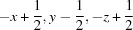
; (ii) 
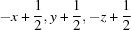
; (iii) 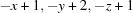; (iv) 
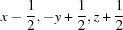
.

**Table 2:** Experimental details

Crystal data	
Chemical formula	C_12_H_10_N_2_·C_8_H_7_NO_4_
*M*_r_	363.36
Crystal system, space group	Monoclinic, *P*2_1_/*n*
Temperature (K)	90
*a*, *b*, *c* (Å)	10.1614 (10), 12.0782 (12), 14.0537 (14)
β (°)	95.027 (2)
*V* (Å^3^)	1718.2 (3)
*Z*	4
Radiation type	Mo *K*α
μ (mm^−1^)	0.10
Crystal size (mm)	0.22 × 0.2 × 0.18

Data collection	
Diffractometer	Bruker *SMART* APEXII CCD
Absorption correction	Multi-scan (*SADABS*; Bruker, 2014 ▸)
*T*_min_, *T*_max_	0.683, 0.747
No. of measured, independent and observed [*I* > 2σ(*I*)] reflections	24372, 6546, 4519
*R*_int_	0.033
(sin θ/λ)_max_ (Å^−1^)	0.771

Refinement	
*R*[*F* ^2^ > 2σ(*F* ^2^)], *wR*(*F* ^2^), *S*	0.047, 0.143, 1.02
No. of reflections	6546
No. of parameters	378
H-atom treatment	H atoms treated by a mixture of independent and constrained refinement
Δρ_max_, Δρ_min_ (e Å^−3^)	0.40, −0.24

Computer programs: *APEX2* and *SAINT* (Bruker, 2014 ▸), *SHELXT* (Sheldrick, 2015 ▸), *SHELXL2014* (Sheldrick, 2015 ▸b) and *OLEX2* (Dolomanov *et al.*, 2009 ▸).

## supplementary crystallographic information

### Crystal data

**Table:**

C_12_H_10_N_2_·C_8_H_7_NO_4_	*F*(000) = 760
*M**_r_* = 363.36	*D*_x_ = 1.405 Mg m^−^^3^
Monoclinic, *P*2_1_/*n*	Mo *K*α radiation, λ = 0.71073 Å
*a* = 10.1614 (10) Å	Cell parameters from 428 reflections
*b* = 12.0782 (12) Å	θ = 2.8–22.0°
*c* = 14.0537 (14) Å	µ = 0.10 mm^−^^1^
β = 95.027 (2)°	*T* = 90 K
*V* = 1718.2 (3) Å^3^	Block, yellow
*Z* = 4	0.22 × 0.2 × 0.18 mm

### Data collection

**Table:**

Bruker SMART APEXII CCD diffractometer	6546 independent reflections
Radiation source: microfocus rotating anode, Incoatec Iµs	4519 reflections with *I* > 2σ(*I*)
Mirror optics monochromator	*R*_int_ = 0.033
Detector resolution: 7.9 pixels mm^-1^	θ_max_ = 33.2°, θ_min_ = 2.2°
ω scans	*h* = −15→15
Absorption correction: multi-scan (*SADABS*; Bruker, 2014)	*k* = −16→18
*T*_min_ = 0.683, *T*_max_ = 0.747	*l* = −19→21
24372 measured reflections	

### Refinement

**Table:**

Refinement on *F*^2^	0 restraints
Least-squares matrix: full	Hydrogen site location: mixed
*R*[*F*^2^ > 2σ(*F*^2^)] = 0.047	H atoms treated by a mixture of independent and constrained refinement
*wR*(*F*^2^) = 0.143	*w* = 1/[σ^2^(*F*_o_^2^) + (0.0727*P*)^2^ + 0.2884*P*] where *P* = (*F*_o_^2^ + 2*F*_c_^2^)/3
*S* = 1.02	(Δ/σ)_max_ < 0.001
6546 reflections	Δρ_max_ = 0.40 e Å^−^^3^
378 parameters	Δρ_min_ = −0.24 e Å^−^^3^

### Special details

**Table:**

Geometry. All esds (except the esd in the dihedral angle between two l.s. planes) are estimated using the full covariance matrix. The cell esds are taken into account individually in the estimation of esds in distances, angles and torsion angles; correlations between esds in cell parameters are only used when they are defined by crystal symmetry. An approximate (isotropic) treatment of cell esds is used for estimating esds involving l.s. planes.

### Fractional atomic coordinates and isotropic or equivalent isotropic displacement parameters (Å^2^)

**Table:**

	*x*	*y*	*z*	*U*_iso_*/*U*_eq_	Occ. (<1)
O1	0.21821 (10)	0.91797 (6)	0.42759 (6)	0.0299 (2)	
O2	0.18838 (9)	0.81890 (6)	0.55840 (5)	0.02654 (18)	
H2	0.1646 (19)	0.8932 (16)	0.5810 (13)	0.058 (5)*	
O3	0.35542 (10)	0.33662 (6)	0.42624 (6)	0.0309 (2)	
O4	0.35891 (9)	0.43117 (6)	0.56369 (5)	0.02640 (18)	
H4	0.388 (2)	0.3606 (16)	0.5933 (14)	0.064 (6)*	
N1	0.27113 (12)	0.63283 (8)	0.17186 (7)	0.0291 (2)	
H1A	0.2656 (17)	0.5692 (14)	0.1385 (12)	0.043 (4)*	
H1B	0.2351 (16)	0.6925 (14)	0.1424 (11)	0.040 (4)*	
C1	0.27076 (11)	0.62966 (8)	0.26981 (7)	0.02010 (19)	
C2	0.29909 (11)	0.53166 (8)	0.32097 (7)	0.02032 (19)	
H2A	0.3164	0.4657	0.2874	0.024*	
C3	0.30223 (10)	0.52951 (8)	0.42012 (7)	0.01921 (19)	
C4	0.27368 (11)	0.62462 (8)	0.47130 (7)	0.01990 (19)	
H4A	0.2728	0.6226	0.5388	0.024*	
C5	0.24644 (10)	0.72289 (8)	0.42033 (7)	0.01881 (19)	
C6	0.24657 (10)	0.72561 (8)	0.32144 (7)	0.01947 (19)	
H6	0.2300	0.7935	0.2884	0.023*	
C7	0.21669 (11)	0.82903 (8)	0.46877 (7)	0.0211 (2)	
C8	0.34070 (11)	0.42315 (8)	0.46985 (7)	0.0215 (2)	
C18	0.89056 (13)	0.89912 (9)	0.43551 (9)	0.0306 (3)	
H18	0.8831	0.9091	0.5019	0.037*	0.588 (3)
H18A	0.8865	0.9167	0.5011	0.037*	0.412 (3)
N2	0.9309 (8)	0.2578 (6)	0.1571 (3)	0.0206 (8)	0.588 (3)
N3	0.8888 (10)	0.9864 (5)	0.3724 (9)	0.0203 (11)	0.588 (3)
C9	0.9283 (3)	0.3491 (2)	0.1046 (2)	0.0259 (5)	0.588 (3)
H9	0.9195	0.3426	0.0370	0.031*	0.588 (3)
C10	0.9382 (2)	0.45426 (16)	0.1453 (2)	0.0246 (4)	0.588 (3)
H10	0.9393	0.5181	0.1058	0.029*	0.588 (3)
C11	0.94634 (18)	0.46501 (16)	0.24375 (18)	0.0202 (4)	0.588 (3)
C12	0.9527 (2)	0.36771 (18)	0.29764 (18)	0.0260 (5)	0.588 (3)
H12	0.9624	0.3709	0.3654	0.031*	0.588 (3)
C13	0.9446 (4)	0.2666 (3)	0.2511 (2)	0.0240 (6)	0.588 (3)
H13	0.9491	0.2008	0.2883	0.029*	0.588 (3)
C14	0.9425 (2)	0.57258 (15)	0.29274 (13)	0.0245 (5)	0.588 (3)
H14	0.9538	0.5724	0.3606	0.029*	0.588 (3)
C15	0.9246 (2)	0.66976 (15)	0.24941 (15)	0.0241 (4)	0.588 (3)
H15	0.9170	0.6692	0.1816	0.029*	0.588 (3)
C16	0.9152 (3)	0.7790 (2)	0.2963 (2)	0.0182 (5)	0.588 (3)
C17	0.9045 (7)	0.7916 (5)	0.3934 (2)	0.0242 (8)	0.588 (3)
H17	0.9064	0.7278	0.4330	0.029*	0.588 (3)
C19	0.9010 (9)	0.9740 (6)	0.2814 (5)	0.0214 (8)	0.588 (3)
H19	0.8997	1.0381	0.2422	0.026*	0.588 (3)
C20	0.9153 (4)	0.8733 (3)	0.2402 (3)	0.0244 (6)	0.588 (3)
H20	0.9254	0.8678	0.1738	0.029*	0.588 (3)
C17A	0.9027 (10)	0.7907 (7)	0.4220 (4)	0.0258 (10)	0.412 (3)
H17A	0.9026	0.7388	0.4728	0.031*	0.412 (3)
C9A	0.9486 (4)	0.3433 (3)	0.0717 (3)	0.0219 (7)	0.412 (3)
H9A	0.9550	0.3259	0.0064	0.026*	0.412 (3)
C20A	0.9161 (6)	0.8429 (4)	0.2607 (4)	0.0260 (11)	0.412 (3)
H20A	0.9275	0.8265	0.1958	0.031*	0.412 (3)
N2A	0.9319 (11)	0.2579 (10)	0.1328 (4)	0.0202 (10)	0.412 (3)
N3A	0.8830 (15)	0.9861 (9)	0.3829 (13)	0.025 (2)	0.412 (3)
C19A	0.8995 (15)	0.9532 (9)	0.2902 (9)	0.034 (2)	0.412 (3)
H19A	0.8996	1.0094	0.2428	0.040*	0.412 (3)
C10A	0.9569 (3)	0.4532 (2)	0.0982 (3)	0.0243 (6)	0.412 (3)
H10A	0.9663	0.5087	0.0514	0.029*	0.412 (3)
C11A	0.9515 (3)	0.4826 (2)	0.1934 (3)	0.0195 (6)	0.412 (3)
C12A	0.9434 (3)	0.3974 (3)	0.2586 (3)	0.0250 (6)	0.412 (3)
H12A	0.9448	0.4125	0.3250	0.030*	0.412 (3)
C13A	0.9330 (6)	0.2880 (4)	0.2245 (4)	0.0293 (10)	0.412 (3)
H13A	0.9262	0.2309	0.2703	0.035*	0.412 (3)
C14A	0.9493 (3)	0.6007 (2)	0.21878 (19)	0.0235 (6)	0.412 (3)
H14A	0.9676	0.6526	0.1709	0.028*	0.412 (3)
C15A	0.9237 (3)	0.6405 (2)	0.3033 (2)	0.0226 (6)	0.412 (3)
H15A	0.9095	0.5884	0.3521	0.027*	0.412 (3)
C16A	0.9155 (4)	0.7585 (4)	0.3276 (3)	0.0186 (7)	0.412 (3)

### Atomic displacement parameters (Å^2^)

**Table:**

	*U*^11^	*U*^22^	*U*^33^	*U*^12^	*U*^13^	*U*^23^
O1	0.0508 (6)	0.0126 (3)	0.0286 (4)	0.0008 (3)	0.0170 (4)	−0.0002 (3)
O2	0.0459 (5)	0.0155 (3)	0.0195 (3)	0.0059 (3)	0.0096 (3)	−0.0016 (3)
O3	0.0510 (6)	0.0143 (3)	0.0270 (4)	0.0041 (3)	0.0012 (4)	−0.0046 (3)
O4	0.0438 (5)	0.0149 (3)	0.0201 (3)	0.0063 (3)	0.0006 (3)	−0.0003 (3)
N1	0.0510 (7)	0.0180 (4)	0.0186 (4)	0.0067 (4)	0.0058 (4)	−0.0018 (3)
C1	0.0246 (5)	0.0175 (4)	0.0187 (4)	0.0010 (4)	0.0046 (4)	−0.0022 (3)
C2	0.0255 (5)	0.0148 (4)	0.0210 (4)	0.0017 (4)	0.0039 (4)	−0.0038 (3)
C3	0.0242 (5)	0.0130 (4)	0.0206 (4)	0.0003 (3)	0.0030 (4)	−0.0012 (3)
C4	0.0269 (5)	0.0142 (4)	0.0191 (4)	0.0002 (4)	0.0047 (4)	−0.0016 (3)
C5	0.0235 (5)	0.0130 (4)	0.0206 (4)	−0.0001 (3)	0.0056 (4)	−0.0026 (3)
C6	0.0236 (5)	0.0140 (4)	0.0213 (4)	0.0010 (3)	0.0050 (4)	−0.0006 (3)
C7	0.0285 (5)	0.0142 (4)	0.0214 (4)	0.0004 (4)	0.0069 (4)	−0.0025 (3)
C8	0.0285 (5)	0.0145 (4)	0.0217 (5)	−0.0002 (4)	0.0025 (4)	−0.0023 (3)
C18	0.0394 (7)	0.0178 (5)	0.0360 (6)	0.0017 (4)	0.0118 (5)	−0.0001 (4)
N2	0.0261 (11)	0.0147 (9)	0.021 (2)	−0.0007 (7)	0.0014 (18)	−0.0088 (18)
N3	0.0306 (19)	0.0099 (16)	0.022 (3)	0.0050 (11)	0.0112 (14)	−0.0001 (12)
C9	0.0351 (14)	0.0194 (9)	0.0238 (13)	−0.0024 (8)	0.0066 (10)	−0.0027 (10)
C10	0.0400 (12)	0.0158 (8)	0.0182 (11)	−0.0012 (7)	0.0044 (9)	−0.0006 (8)
C11	0.0232 (9)	0.0158 (10)	0.0219 (11)	−0.0012 (6)	0.0037 (7)	−0.0024 (7)
C12	0.0388 (12)	0.0170 (9)	0.0225 (10)	0.0016 (8)	0.0038 (9)	−0.0012 (8)
C13	0.0341 (13)	0.0160 (13)	0.0225 (15)	0.0007 (10)	0.0055 (12)	−0.0034 (9)
C14	0.0348 (11)	0.0173 (9)	0.0218 (8)	0.0012 (7)	0.0053 (7)	−0.0064 (6)
C15	0.0325 (10)	0.0176 (8)	0.0223 (9)	−0.0004 (7)	0.0021 (7)	−0.0056 (7)
C16	0.0226 (9)	0.0109 (14)	0.0211 (13)	0.0001 (8)	0.0021 (10)	0.0004 (10)
C17	0.0346 (13)	0.0133 (9)	0.026 (2)	0.0011 (8)	0.0086 (18)	−0.0038 (17)
C19	0.0301 (15)	0.0192 (19)	0.0155 (15)	0.0019 (14)	0.0054 (11)	−0.0047 (15)
C20	0.0324 (12)	0.0204 (16)	0.0205 (14)	0.0012 (12)	0.0019 (10)	−0.0034 (10)
C17A	0.0330 (18)	0.0195 (15)	0.027 (3)	0.0003 (12)	0.011 (3)	−0.005 (2)
C9A	0.0281 (17)	0.0136 (11)	0.0247 (18)	−0.0015 (10)	0.0065 (14)	−0.0010 (13)
C20A	0.0378 (18)	0.019 (3)	0.021 (2)	0.0028 (19)	0.0022 (17)	−0.0085 (17)
N2A	0.0234 (15)	0.0201 (13)	0.017 (3)	0.0010 (10)	−0.001 (2)	−0.010 (2)
N3A	0.026 (3)	0.033 (4)	0.016 (3)	−0.001 (2)	0.008 (2)	−0.011 (2)
C19A	0.039 (3)	0.029 (4)	0.032 (3)	−0.002 (3)	0.0015 (19)	0.006 (2)
C10A	0.0320 (15)	0.0166 (11)	0.0243 (15)	−0.0019 (10)	0.0035 (12)	−0.0024 (10)
C11A	0.0250 (13)	0.0163 (11)	0.0170 (15)	−0.0012 (9)	0.0008 (10)	−0.0038 (11)
C12A	0.0386 (17)	0.0155 (16)	0.0209 (15)	0.0010 (11)	0.0017 (12)	−0.0020 (11)
C13A	0.041 (2)	0.019 (2)	0.027 (3)	0.0004 (15)	0.0012 (19)	0.0024 (15)
C14A	0.0299 (14)	0.0125 (10)	0.0283 (13)	−0.0003 (9)	0.0033 (10)	−0.0035 (9)
C15A	0.0308 (14)	0.0119 (12)	0.0251 (14)	0.0007 (9)	0.0020 (10)	−0.0013 (9)
C16A	0.0207 (13)	0.0107 (14)	0.024 (2)	0.0002 (10)	0.0012 (14)	0.0033 (15)

### Geometric parameters (Å, º)

**Table:**

O1—C7	1.2209 (12)	C12—H12	0.9500
O2—H2	0.990 (19)	C12—C13	1.385 (4)
O2—C7	1.3222 (12)	C13—H13	0.9500
O3—C8	1.2274 (12)	C14—H14	0.9500
O4—H4	0.98 (2)	C14—C15	1.327 (3)
O4—C8	1.3191 (12)	C15—H15	0.9500
N1—H1A	0.899 (17)	C15—C16	1.482 (3)
N1—H1B	0.894 (17)	C16—C17	1.387 (4)
N1—C1	1.3775 (13)	C16—C20	1.385 (3)
C1—C2	1.4017 (14)	C17—H17	0.9500
C1—C6	1.4005 (13)	C19—H19	0.9500
C2—H2A	0.9500	C19—C20	1.360 (7)
C2—C3	1.3912 (14)	C20—H20	0.9500
C3—C4	1.3991 (13)	C17A—H17A	0.9500
C3—C8	1.4979 (14)	C17A—C16A	1.399 (6)
C4—H4A	0.9500	C9A—H9A	0.9500
C4—C5	1.4014 (13)	C9A—N2A	1.361 (10)
C5—C6	1.3902 (14)	C9A—C10A	1.379 (4)
C5—C7	1.4948 (13)	C20A—H20A	0.9500
C6—H6	0.9500	C20A—C19A	1.410 (11)
C18—H18	0.9500	C20A—C16A	1.388 (5)
C18—H18A	0.9500	N2A—C13A	1.338 (7)
C18—N3	1.376 (9)	N3A—C19A	1.39 (2)
C18—C17	1.439 (5)	C19A—H19A	0.9500
C18—C17A	1.331 (9)	C10A—H10A	0.9500
C18—N3A	1.283 (13)	C10A—C11A	1.390 (5)
N2—C9	1.326 (7)	C11A—C12A	1.385 (4)
N2—C13	1.320 (5)	C11A—C14A	1.472 (4)
N3—C19	1.305 (13)	C12A—H12A	0.9500
C9—H9	0.9500	C12A—C13A	1.406 (6)
C9—C10	1.392 (3)	C13A—H13A	0.9500
C10—H10	0.9500	C14A—H14A	0.9500
C10—C11	1.386 (3)	C14A—C15A	1.329 (4)
C11—C12	1.397 (3)	C15A—H15A	0.9500
C11—C14	1.472 (2)	C15A—C16A	1.469 (5)
			
C7—O2—H2	107.5 (11)	C15—C14—C11	125.02 (18)
C8—O4—H4	111.7 (11)	C15—C14—H14	117.5
H1A—N1—H1B	116.4 (15)	C14—C15—H15	116.7
C1—N1—H1A	119.4 (11)	C14—C15—C16	126.5 (2)
C1—N1—H1B	116.6 (10)	C16—C15—H15	116.7
N1—C1—C2	121.17 (9)	C17—C16—C15	123.3 (3)
N1—C1—C6	120.72 (9)	C20—C16—C15	118.4 (3)
C6—C1—C2	118.07 (9)	C20—C16—C17	118.3 (4)
C1—C2—H2A	119.5	C18—C17—H17	119.2
C3—C2—C1	121.01 (9)	C16—C17—C18	121.5 (4)
C3—C2—H2A	119.5	C16—C17—H17	119.2
C2—C3—C4	120.80 (9)	N3—C19—H19	118.6
C2—C3—C8	117.74 (8)	N3—C19—C20	122.9 (6)
C4—C3—C8	121.44 (9)	C20—C19—H19	118.6
C3—C4—H4A	120.9	C16—C20—H20	120.4
C3—C4—C5	118.24 (9)	C19—C20—C16	119.2 (4)
C5—C4—H4A	120.9	C19—C20—H20	120.4
C4—C5—C7	122.17 (9)	C18—C17A—H17A	122.4
C6—C5—C4	120.90 (9)	C18—C17A—C16A	115.3 (5)
C6—C5—C7	116.93 (8)	C16A—C17A—H17A	122.4
C1—C6—H6	119.5	N2A—C9A—H9A	117.7
C5—C6—C1	120.93 (9)	N2A—C9A—C10A	124.5 (5)
C5—C6—H6	119.5	C10A—C9A—H9A	117.7
O1—C7—O2	123.09 (9)	C19A—C20A—H20A	120.4
O1—C7—C5	121.82 (9)	C16A—C20A—H20A	120.4
O2—C7—C5	115.09 (8)	C16A—C20A—C19A	119.2 (6)
O3—C8—O4	123.31 (9)	C13A—N2A—C9A	114.3 (9)
O3—C8—C3	122.38 (9)	C18—N3A—C19A	107.5 (10)
O4—C8—C3	114.30 (8)	C20A—C19A—H19A	117.5
N3—C18—H18	122.5	N3A—C19A—C20A	125.0 (9)
N3—C18—C17	115.0 (5)	N3A—C19A—H19A	117.5
C17—C18—H18	122.5	C9A—C10A—H10A	120.1
C17A—C18—H18A	111.8	C9A—C10A—C11A	119.9 (3)
N3A—C18—H18A	111.8	C11A—C10A—H10A	120.1
N3A—C18—C17A	136.5 (8)	C10A—C11A—C14A	118.9 (3)
C13—N2—C9	119.0 (5)	C12A—C11A—C10A	117.2 (2)
C19—N3—C18	123.1 (6)	C12A—C11A—C14A	123.8 (3)
N2—C9—H9	118.9	C11A—C12A—H12A	120.7
N2—C9—C10	122.3 (3)	C11A—C12A—C13A	118.6 (3)
C10—C9—H9	118.9	C13A—C12A—H12A	120.7
C9—C10—H10	120.3	N2A—C13A—C12A	125.3 (6)
C11—C10—C9	119.4 (2)	N2A—C13A—H13A	117.4
C11—C10—H10	120.3	C12A—C13A—H13A	117.4
C10—C11—C12	117.31 (16)	C11A—C14A—H14A	117.4
C10—C11—C14	123.2 (2)	C15A—C14A—C11A	125.2 (3)
C12—C11—C14	119.4 (2)	C15A—C14A—H14A	117.4
C11—C12—H12	120.4	C14A—C15A—H15A	117.3
C13—C12—C11	119.2 (2)	C14A—C15A—C16A	125.3 (3)
C13—C12—H12	120.4	C16A—C15A—H15A	117.3
N2—C13—C12	122.7 (4)	C17A—C16A—C15A	120.1 (5)
N2—C13—H13	118.6	C20A—C16A—C17A	116.4 (5)
C12—C13—H13	118.6	C20A—C16A—C15A	123.5 (4)
C11—C14—H14	117.5		
			
N1—C1—C2—C3	178.19 (10)	C11—C14—C15—C16	177.6 (2)
N1—C1—C6—C5	−179.82 (10)	C12—C11—C14—C15	−173.6 (2)
C1—C2—C3—C4	1.71 (16)	C13—N2—C9—C10	−0.7 (9)
C1—C2—C3—C8	−176.64 (10)	C14—C11—C12—C13	174.4 (3)
C2—C1—C6—C5	−2.05 (16)	C14—C15—C16—C17	−10.0 (5)
C2—C3—C4—C5	−2.20 (16)	C14—C15—C16—C20	170.8 (3)
C2—C3—C8—O3	−7.84 (17)	C15—C16—C17—C18	−177.6 (3)
C2—C3—C8—O4	171.27 (10)	C15—C16—C20—C19	177.1 (5)
C3—C4—C5—C6	0.59 (16)	C17—C18—N3—C19	−1.1 (12)
C3—C4—C5—C7	−179.15 (10)	C17—C18—C17A—C16A	1.4 (17)
C4—C3—C8—O3	173.81 (11)	C17—C18—N3A—C19A	−3.8 (15)
C4—C3—C8—O4	−7.07 (15)	C17—C16—C20—C19	−2.0 (7)
C4—C5—C6—C1	1.56 (16)	C20—C16—C17—C18	1.5 (7)
C4—C5—C7—O1	165.59 (11)	C17A—C18—N3—C19	−0.5 (14)
C4—C5—C7—O2	−14.70 (15)	C17A—C18—C17—C16	−179 (3)
C6—C1—C2—C3	0.43 (16)	C17A—C18—N3A—C19A	−4.3 (18)
C6—C5—C7—O1	−14.15 (16)	C9A—N2A—C13A—C12A	3.1 (13)
C6—C5—C7—O2	165.55 (10)	C9A—C10A—C11A—C12A	2.7 (4)
C7—C5—C6—C1	−178.69 (10)	C9A—C10A—C11A—C14A	−174.9 (3)
C8—C3—C4—C5	176.09 (10)	N2A—C9A—C10A—C11A	1.6 (8)
C18—N3—C19—C20	0.6 (15)	N3A—C18—N3—C19	−159 (11)
C18—C17A—C16A—C20A	0.9 (9)	N3A—C18—C17—C16	2.5 (10)
C18—C17A—C16A—C15A	−177.3 (5)	N3A—C18—C17A—C16A	2.8 (15)
C18—N3A—C19A—C20A	2 (2)	C19A—C20A—C16A—C17A	−2.1 (11)
N2—C9—C10—C11	−2.3 (6)	C19A—C20A—C16A—C15A	176.0 (8)
N3—C18—C17—C16	0.0 (8)	C10A—C9A—N2A—C13A	−4.4 (12)
N3—C18—C17A—C16A	−0.3 (12)	C10A—C11A—C12A—C13A	−3.8 (5)
N3—C18—N3A—C19A	19 (9)	C10A—C11A—C14A—C15A	168.9 (3)
N3—C19—C20—C16	1.0 (12)	C11A—C12A—C13A—N2A	0.9 (9)
C9—N2—C13—C12	1.9 (9)	C11A—C14A—C15A—C16A	−177.1 (3)
C9—C10—C11—C12	4.0 (3)	C12A—C11A—C14A—C15A	−8.5 (5)
C9—C10—C11—C14	−173.2 (2)	C14A—C11A—C12A—C13A	173.6 (4)
C10—C11—C12—C13	−2.9 (3)	C14A—C15A—C16A—C17A	−172.8 (5)
C10—C11—C14—C15	3.5 (3)	C14A—C15A—C16A—C20A	9.1 (6)
C11—C12—C13—N2	−0.1 (6)	C16A—C20A—C19A—N3A	0.5 (19)

### Hydrogen-bond geometry (Å, º)

**Table:**

*D*—H···*A*	*D*—H	H···*A*	*D*···*A*	*D*—H···*A*
N1—H1*A*···O1^i^	0.899 (17)	2.062 (17)	2.9540 (13)	171.0 (15)
N1—H1*B*···O3^ii^	0.894 (17)	2.157 (17)	3.0500 (13)	178.6 (13)
O2—H2···N3^iii^	0.989 (19)	1.70 (2)	2.688 (8)	173.4 (18)
O2—H2···N3*A*^iii^	0.989 (19)	1.63 (2)	2.619 (12)	177 (2)
O4—H4···N2^iv^	0.98 (2)	1.72 (2)	2.702 (7)	173.2 (19)
O4—H4···N2*A*^iv^	0.98 (2)	1.59 (2)	2.566 (11)	175 (2)

Symmetry codes: (i) −*x*+1/2, *y*−1/2, −*z*+1/2; (ii) −*x*+1/2, *y*+1/2, −*z*+1/2; (iii) −*x*+1, −*y*+2, −*z*+1; (iv) *x*−1/2, −*y*+1/2, *z*+1/2.
